# Secretory carcinoma of the breast: A case report

**DOI:** 10.1016/j.ijscr.2019.02.029

**Published:** 2019-02-28

**Authors:** Kamil Pohlodek, Iveta Mečiarová, Petr Grossmann, Petr Martínek, Zdeněk Kinkor

**Affiliations:** a 2nd Department of Gynecology and Obstetrics, Faculty of Medicine, Comenius University of Bratislava, Slovakia; b Alpha Medical Pathology Ltd., Bratislava, Slovakia; c Department of Pathology, Charles University in Prague, Faculty of Medicine in Plzen, Czech Republic; d Bioptic Laboratory, Ltd., Plzen, Czech Republic

**Keywords:** Breast cancer, Breast imaging, Breast surgery, Sectretory carcinoma, Immunohistochemistry, Fluorescence in situ hybridization, Case report

## Abstract

**Introduction:**

Secretory breast carcinoma (SBC) is a rare breast tumor which accounts for < 0.15% of all breast cancers. It was originally described as a juvenile breast carcinoma, occurring in young children and adolescent women. SBC is associated with a characteristic balanced translocation, t(12;15), that creates a*ETV6-NTRK3* gene fusion.

**Presentation of case:**

A 52-year-old Caucasian woman had palpable lump in her right breast. After breast imaging examination (BI-RADS 4b) and preoperative core-needle biopsy with suspicion of SBC a breast conserving therapy was performed. The diagnosis of SBC was confirmed through immunohistochemistry and cytogenetic examination of the tumor. The patient is now 22 months post‑surgery and remains disease‑free.

**Discussion:**

Recent studies reported that the disease occurs at a later age than previously recognized, and is associated with good long-term survival. In breast imaging it may mimic a benign tumor. Immunohistochemistry and cytogenetic analysis of the tumor are crucial for confirmation of SBC.

**Conclusion:**

There is no consensus with regard to the best treatment strategy for patients with SBC. Breast conserving therapy with sentinel lymph nodes biopsy is at present the first choice treatment. Further research for a specific NTRK3 tyrosine kinase inhibitor could lead to the discovery of a new targeted treatment of this tumor.

## Highlights

• Secretory breast carcinoma is a rare tumor which accounts for < 0.15% of all breast cancers
• In breast imaging it may mimic a benign tumor.
• SBC is associated with a balanced translocation, t(12;15), that creates a *ETV6NTRK3* gene fusion.
• There is no consensus with regard to the best treatment strategy for patients with SBC.
• Further research could lead to the discovery of a new targeted treatment of this tumor

## 1 Introduction

WHO has defined the secretory breast carcinoma (SBC) as „a rare, low-grade, translocation-associated invasive carcinoma with a solid, microcystic and tubular architecture composed of cells that produce intracellular and extracellular secretory material “and classed it between „exceptionally rare types and variants “of breast tumors [1]. SBCs account for < 0.15% of all breast cancers [2]. Although it was originally described as a juvenile breast carcinoma, occurring in young children, most cases have been reported in adults of both sexes with the median age of presentation in 25 years [3]. SBC is associated with a characteristic balanced translocation, t(12;15), that creates a *ETV6-NTRK3* gene fusion [[4], [5], [6]]. Typically, secretory breast carcinomas are negative for hormone receptors and do not express human epidermal growth factor receptor 2 (HER2) [7]. The breast imaging characteristics of SBC are non-specific and can mimic benign breast tumor. A definitive diagnosis of SBC is established from histology, immunohistochemistry and cytogenetic evaluation. We present in our study a patient with SBC in line with the SCARE criteria [8].

## 2 Case report

**Fig. 1:**
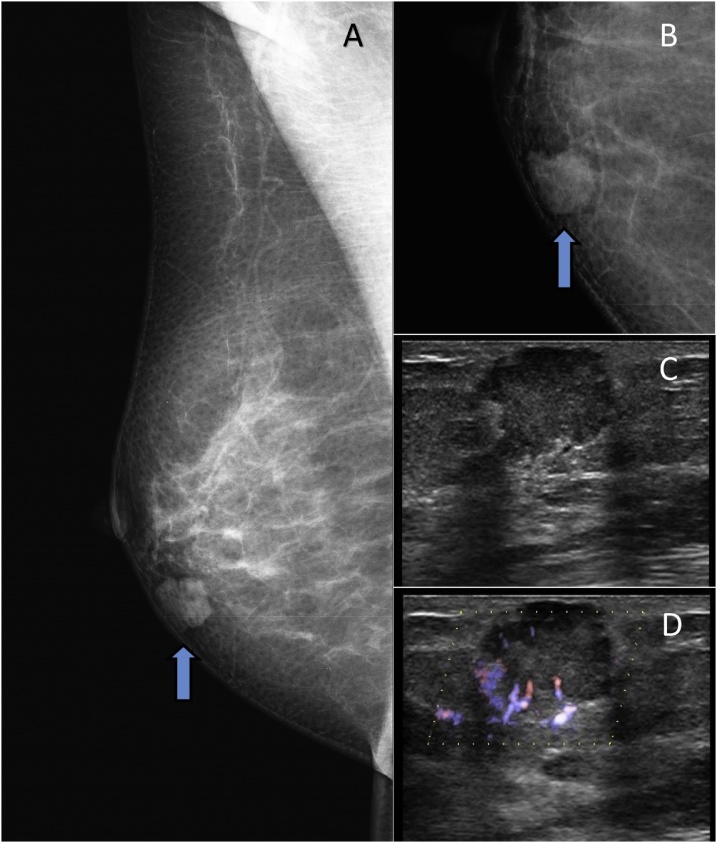
Breast imaging. Mammography suggested a lobulated, radiopaque lesion with mostly sharp contours and hallo sign (A,B). Breast ultrasonography revealed an round-shaped, low-echoic tumor of unclear etiology (C) with a pathological pattern of blood flow, as seen on Power-Doppler imaging (D).

A 52-year-old Caucasian woman with no significant previous medical history was referred to the Breast Unit of the 2nd Department of OB/GYN, University Hospital of Bratislava, Slovakia, for assessment of a palpable lump in her right breast. Physical examination revealed a superficial, 1.5‑cm lump, located in the lower, inner quadrant of the right breast with clinically negative axillary lymph nodes. Mammography suggested a lobulated, radiopaque 1.5-cm lesion with mostly sharp contours and halo sign (Fig. 1A,B). Breast ultrasonography revealed an round-shaped, low-echoic tumor of unclear etiology (Fig. 1C) with a pathological pattern of blood flow, as seen on Power-Doppler imaging (Fig. 1D). The results of breast imaging examinations were classified as BI-RADS 4b. Infiltrating ductal carcinoma could not be excluded. A ultrasound-guided large-core-needle biopsy was performed and histologic diagnosis of a SBC was supposed. The patient underwent breast conserving surgery with sentinel lymph node biopsy.

**Fig. 2:**
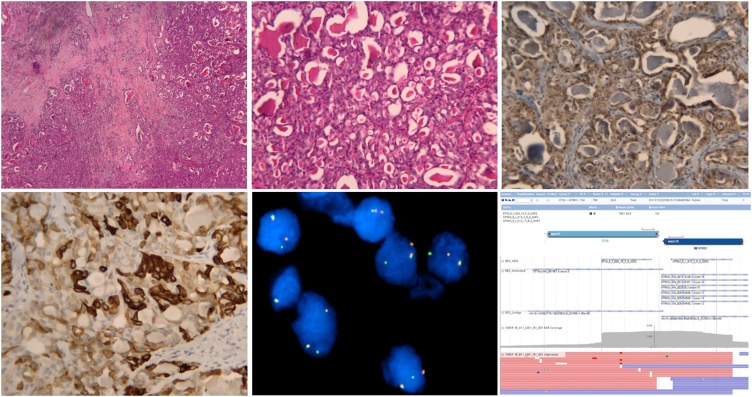
Histologic and cytogenetic evaluation. The tumor is composed of admixture of microcystic, ductal and solid patterns (A). Histologic hallmark is the presence of abundant intra- and extracellular dense pink PAS positive secretions, especially in microcystic extracellular spaces (B). Immunohistochemisty showed positive results for c-Kit protein (C), and cytokeratins 5,14 (D). FISH analysis by break-apart probe showed detected break of gene *NTRK3* (15q25.3). Fusion of yellow (orange-yellow-green) signals demonstrates one of an intact copy of gene *NTRK3*, split orange and green signals indicate a break in the second copy of *NTRK3* gene (E). Schematic visualisation of detected fusion transcript *ETV6-NTRK3* together with the annotation of fusion partners and information about the depth of reading of studied area including particular reads (F).

Histological evaluation of the surgical specimens showed monoform tumor cells with eosinophilic cytoplasm with vacuoli and thyroid-like pseudofolicles, round nuclei with intermediate mitotic activity (MAI 10 MF/10 HPF) (Fig. 2A). The tumor presented with focally infiltrative growth without in situ component. The immunohistochemistry (IHC) showed periodic acid–Schiff (PAS) positive secretory material in intra- and extracellular spaces (Fig. 2B) and negative results for estrogen, progesteron and HER2 receptors. The tumor was positive for cytokeratins 5, 14 and c-Kit protein (CD117) (Fig. 2C,D). There was also diffuse IHC positivity for S100, MUC4, EMA, and scattered positivity for gross cystic disease fluid protein 15. Pan-TRK staining was provided to detect possible neurotrophic tyrosine receptor kinase (NTRK) fusions. Resections margins of more than 10 mm were tumor-free and two sentinel lymph nodes were found to be free of metastases. A dual color break apart probe (SureFISH®, Agilent, St.Clara, USA) was used for fluorescence in situ hybridization (FISH) evaluation of *NTRK3* (15q25.3) gene (Fig. 2E). The *ETV6-NTRK3* gene fusion was confirmed through FusionPlex® assay kit for solid tumors (ArcherDX, Inc, Colorado, USA). Next generation sequencing (NGS) analysis was used for schematic visualisation of detected fusion transcript *ETV6-NTRK3* together with information about the depth of reading of studied area (Fig. 2F).

The post‑operative course was uneventful and the patient was discharged home on post‑operative day 4. The patient received adjuvant radiation therapy with a total dose of 50 Gy in 26 fractions. She now 22 months post‑surgery and remains disease‑free.

## 3 Discussion

SBC has a low-grade clinical course and is associated with a favourable prognosis [3,7,9]. It was originally described in children and adolescent women, with a characteristic morphology and controversy regarding the choice of treatment. Wang et al. [10] refered about a 12-year-old female who presented with SBC in her left breast and underwent breast-conserving therapy without reccurence of the disease. Herz et al. [11] presented case of a 27-year-old woman with pulmonary metastases from a secretory breast cancer treated by mastectomy and axillary lymph node dissection. There was no response to chemotherapy; she died of respiratory failure two and a half years after presentation. Li et al. [12] recently refered about clinical features and treatment of male SBC. A rare occurrence in the ectopic breast tissue of the axilla has been also reported [13].

In mammography, SBC usually presents as a distinctly spiculated lesion or as a discrete, lobulated, solitary mass with smooth or irregular borders, which may mimic a fibroadenoma [7,9]. The ultrasound findings also mimic benign lesions, demonstrating a well-circumscribed, hypoechoic to isoechoic mass, sometimes with microlobulations [14]. The lesion is usually homogeneous, but it can be heterogeneous [7,11]. In SBC three morphologic patterns are seen in various combinations: microcystic, solid and tubular [1]. The microcystic pattern is composed of small cysts mimicking thyroid follicles. The tubular pattern shows lumina containing secretions. The presence of intra- and extracellular secretory, PAS-positive material (Fig. 2B) is a consistent finding [[1], [2], [3]]. SBCs are in most cases triple negative [3,6,9].

SBC is associated with a characteristic balanced translocation, t(12;15) (p13;q25). This translocation is known to be oncogenic also in another types of neoplasia [[4], [5], [6]]. It affects genes *ETV6 (TEL)* on the chromosome 12 and *NTRK3 (TRKC)* on the chromosome 15. The most often it comes to the breakage in the intron 5 of the gene *ETV6* and the intron 15 of the gene *NTRK3* (Fig. 2F). This creates a fusion gene; or more precisely a protein, where there is N-terminal helix-loop-helix (HLH) domain of the highly expressed transcription factor ETV6 linked to tyrosine kinase domain of the gene *NTRK3*. The chimeric protein is subject to the ligand dependent HLH mediated dimerization with a subsequent activation of NTRK3 tyrosine kinase domain. The activated tyrosine kinase, then, through a number of signaling pathways, acts in the cell transformation [3,6]. The differential diagnosis with acinic carcinoma is based on the absence of the *ETV6-NTRK3* translocation in acinic carcinomas.

Up-to-date, there is no consensus with regard to the best treatment strategy for patients with SBC. At present, surgical excision is the primary therapy for these cases. Axillary metastasis is rare, particularly if the tumor is <2 cm like in this case. Thus, conservative treatment without lymph node examination has been frequently proposed. However, axillary lymph node metastasis has been reported from a 1.5-cm secretory tumor. Involvement of more than three lymph nodes may indicate a risk for distant metastasis and a poor outcome. Therefore, examination of lymph node status using a sentinel lymph node biopsy or axillary lymph node dissection should be performed [9,15]. In general, adjuvant radiation therapy following breast conserving surgery improves loco regional control and disease-specific survival. Although there has been only few reports regarding the effectiveness of radiation therapy in SBC, adjuvant radiation therapy may improve long-term survival as it does for other types of invasive breast cancer. The use of adjuvant chemotherapy has not been thoroughly investigated in SBC, moreover systemic metastasis is rare. Horowitz et al [15] refered in their analysis of SEER data from 83 patients with SBC diagnosed between the years 1983 and 2007 that SBC occurs at a later age than previously recognized, and is associated with good long-term survival.

## 4 Conclusion

SBC is a rare and indolent type of breast tumor. It presents usually as a palpable breast mass and may resemble a benign lesion using breast imaging. SBCs are slow‑growing tumors and generally have a good prognosis. Radiologists should be aware of this rare tumor's nonspecific imaging features and clinical implications when making a differential diagnosis of solid breast masses. Breast conserving therapy with/without adjuvant chemotherapy is at present the standart treatment of SBC. Generally, the tumors are triple negative, so hormonal treatment or targeted anti-HER2 therapies are not considered in most cases. Because of characteristic balanced translocation t(12;15) in SBC, further research for a specific NTRK3 tyrosine kinase inhibitor could lead to the discovery of a new targeted treatment of this tumor [16,17].

## Conflicts of interest

The authors herewith disclose no financial and personal relationships with other people or organisations that could inappropriately influence this work.

The authors declare no conflict of interest for writing this manuscript.

## Sources of funding

No funding.

## Ethical approval

The case report was approved by institutional review board.

## Consent

The patient signed written informed consent for the publication.

## Author contribution

Kamil Pohlodek: Conceptualization, Visualization, Investigation, Data curation, Writing- Original draft preparation.

Iveta Mečiarová: Histopahological examination.

Petr Martínek: Cytogenetic examinations.

Petr Grossmann: Supervision of cytogenetic examinations.

Zdeněk Kinkor: Supervision of histopathological examinations

## Registration of research studies

In accordance with the Declaration of Helsinki 2013, all research involving human participants has to be registered in a publicly accessible database.  Please enter the name of the registry and the unique identifying number (UIN) of your study.

You can register any type of research at http://www.researchregistry.com to obtain your UIN if you have not already registered. This is mandatory for human studies only.  Trials and certain observational research can also be registered elsewhere such as: ClinicalTrials.gov or ISRCTN or numerous other registries.

## Guarantor

Kamil Pohlodek, MD, PhD

Professor of OB/GYN

University Hospital of Bratislava

Corresponding author

on behalf of all co-authors

kamil.pohlodek@fmed.uniba.sk

phone: +421 905 311566

## Provenance and peer review

Not commissioned, externally peer reviewed.
